# CG Dinucleotide Removal in Bioluminescent and Fluorescent Reporters Improves HIV-1 Replication and Reporter Gene Expression for Dual Imaging in Humanized Mice

**DOI:** 10.1128/JVI.00449-21

**Published:** 2021-09-09

**Authors:** Chandra N. Roy, Mariana A. Benitez Moreno, Chris Kline, Zandrea Ambrose

**Affiliations:** a Department of Microbiology and Molecular Genetics, University of Pittsburgh School of Medicine, Pittsburgh, Pennsylvania, USA; b Department of Infectious Diseases and Microbiology, University of Pittsburgh Graduate School of Public Health, Pittsburgh, Pennsylvania, USA; Icahn School of Medicine at Mount Sinai

**Keywords:** animal models, antiretroviral therapy, human immunodeficiency virus, *in vivo* imaging, reporter viruses, viral reservoir

## Abstract

Visualizing the transmission and dissemination of human immunodeficiency virus type 1 (HIV-1) in real time in humanized mouse models is a robust tool to investigate viral replication during treatments and in tissue reservoirs. However, the stability and expression of HIV-1 reporter genes are obstacles for long-term serial imaging *in vivo*. Two replication-competent CCR5-tropic HIV-1 reporter constructs were created that encode either nanoluciferase (nLuc) or a near*-*infrared fluorescent protein (iRFP) upstream of *nef*. HIV-1 reporter virus replication and reporter gene expression was measured in cell culture and in humanized mice. While reporter gene expression *in vivo* correlated initially with plasma viremia, expression decreased after 4 to 5 weeks despite high plasma viremia. The reporter genes were codon optimized to remove cytosine/guanine (CG) dinucleotides, and new CO-nLuc and CO-iRFP viruses were reconstructed. Removal of CG dinucleotides in HIV-1 reporter viruses improved replication *in vitro* and reporter expression *in vivo* and *ex vivo*. Both codon-optimized reporter viruses could be visualized during coinfection and *in vivo* reporter gene expression during treatment failure preceded detection of plasma viremia. While the dynamic range of CO-iRFP HIV-1 was lower than that of CO-nLuc HIV-1, both viruses could have utility in studying and visualizing HIV-1 infection in humanized mice.

**IMPORTANCE** Animal models are important for studying HIV-1 pathogenesis and treatments. We developed two viruses each encoding a reporter gene that can be expressed in cells after infection. This study shows that HIV-1 infection can be visualized by noninvasive, whole-body imaging in mice with human immune cells over time by reporter expression. We improved reporter expression to reflect HIV-1 replication and showed that two viral variants can be tracked over time in the same animal and can predict failure of antiretroviral therapy to suppress virus.

## INTRODUCTION

Animals models for human immunodeficiency virus type 1 (HIV-1) transmission, infection, and pathogenesis, primarily nonhuman primates and mice with humanized immune systems (HIS mice), have provided important advances in developing prevention methods (1, 2) and therapies (3), as well as improving our understanding on viral reservoirs and potential HIV-1 eradication strategies (4). Animal models allow controlled infections and interventions with defined viruses and sampling time points that are often variable in human populations. In addition, tissues, including those unable to be biopsied in live humans, can be interrogated for drug concentrations, frequency of infected cells, immune responses, and other virologic or immunologic parameters.

Recent use of noninvasive whole animal imaging of simian immunodeficiency virus (SIV) using antibody-targeted positron emission tomography (PET) in nonhuman primates (5, 6) or HIV-1 encoding the nanoluciferase (nLuc) gene in HIS mice (7) has provided spatiotemporal assessment of infection *in vivo*. Using these techniques, virus dissemination, the efficacy of prophylactic therapies or vaccines and treatment, and the reversal of latency potentially can be visualized with relative ease. Imaging infection over time in animal models has advantages over traditional virology assays. For example, whole-animal imaging of HIV-1 can interrogate multiple anatomic compartments and is more rapid than performing quantitative PCR/RT-PCR.

To study HIV-1 dissemination in HIS mice using *in vivo* imaging, we produced a replication-competent CCR5-tropic HIV-1 that encodes nLuc, similar to what has previously been described (7). Since bioluminescent imaging requires a substrate for *in vivo* imaging or antibodies for *ex vivo* imaging, we also developed HIV-1 encoding the near-infrared fluorescent protein iRFP670 (iRFP) that could be visualized in whole animals and at the cellular level in the absence of administration of a substrate. However, nLuc and iRFP expression in HIS mice decreased 4 to 5 weeks postinfection despite stable plasma viremia. Codon optimization by removal of cytosine/guanine (CG) dinucleotides in the reporter genes led to higher *in vitro* HIV-1 replication and improved correlation of *in vivo* and *ex vivo* reporter gene expression with plasma viremia. Infection of mice with both codon-optimized reporter viruses was successful.

## RESULTS

### Replication and expression of reporter viruses in CD4^+^ T cells.

**FIG 1:**
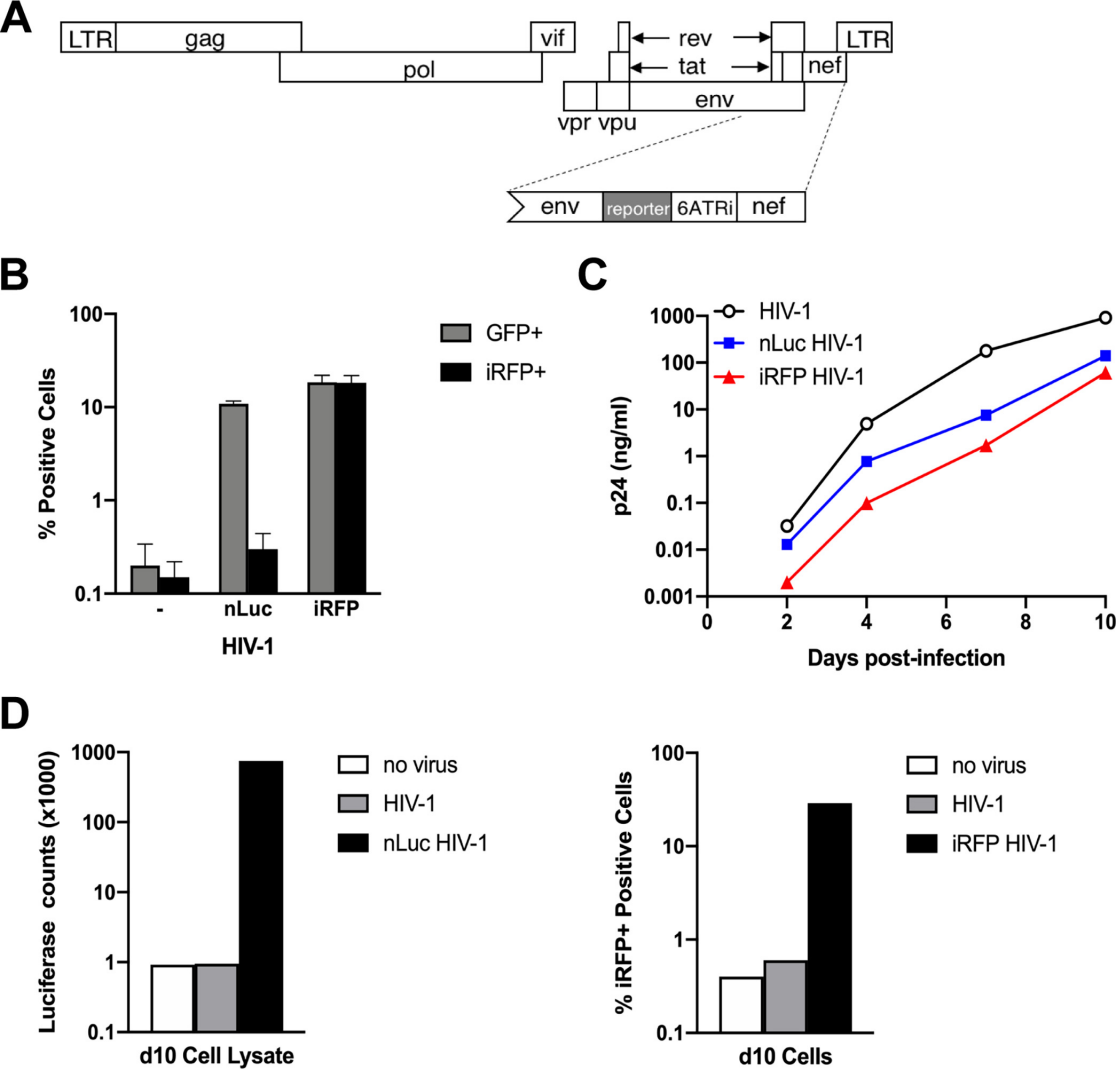
Development and *in vitro* characterization of replication-competent HIV-1 reporter viruses. (A) A schematic of the HIV-1 genome shows the reporter gene (nLuc or iRFP) and the 6ATRi sequence between *env* and *nef*. (B) Infectivity of nLuc HIV-1 and iRFP HIV-1 stocks (0.1 μl) in the GHOST reporter cell line was measured by flow cytometry for GFP and iRFP expression. Error bars represent standard deviations (SD) of duplicate infections. (C) HuT-R5 cells were infected with equal MOI of wild-type HIV-1, nLuc HIV-1, or iRFP HIV-1. Replication was measured by p24 production in the cell supernatant over time. (D) nLuc (left) or iRFP (right) expression was measured in HuT-R5 cells taken at day 10 from cultures shown in panel C.

To visualize HIV-infected cells *in vivo*, the CCR5-tropic, replication-competent HIV-1_NL4-BAL_ strain was engineered to express either nLuc or iRFP (nLuc HIV-1 or iRFP HIV-1, respectively; Fig. 1A), similar to a previously described construct (8). The reporter genes were introduced upstream of the encephalomyocarditis virus internal ribosome entry site (IRES), 6ATRi, to allow expression of Nef. Reporter viruses were produced, and an equal volume of each virus was used to infect lentivirus reporter GHOST cells, human osteosarcoma cells expressing CD4 and HIV-1 coreceptors that also contain a green fluorescent protein (GFP) expression cassette under the HIV-2 long-terminal repeat (LTR) (9). The frequency of cells expressing GFP was similar for both viruses and, for iRFP HIV-1, the frequency of cells expressing iRFP was similar to the frequency of GFP^+^ cells (Fig. 1B). Replication was investigated for both reporter viruses, as well as HIV-1 lacking a reporter gene (wild-type HIV-1) at equal multiplicities of infection (MOI) using HuT-R5 cells, a CD4^+^ T cell line expressing CCR5 (10). Although all viruses replicated with similar kinetics, reporter virus production, particularly for iRFP HIV-1, was reduced compared to WT HIV-1 (Fig. 1C). At day 10 postinfection, HuT-R5 cells from the cultures were evaluated for nLuc and iRFP expression. Cells infected with nLuc HIV-1 or iRFP HIV-1 had nearly 1,000-fold higher nLuc or 30-fold higher iRFP expression over background (Fig. 1D). These results suggested that both reporter viruses replicate well and express their reporter genes for at least 10 days *in vitro*.

### Replication and expression of reporter viruses in HIS mice.

**FIG 2:**
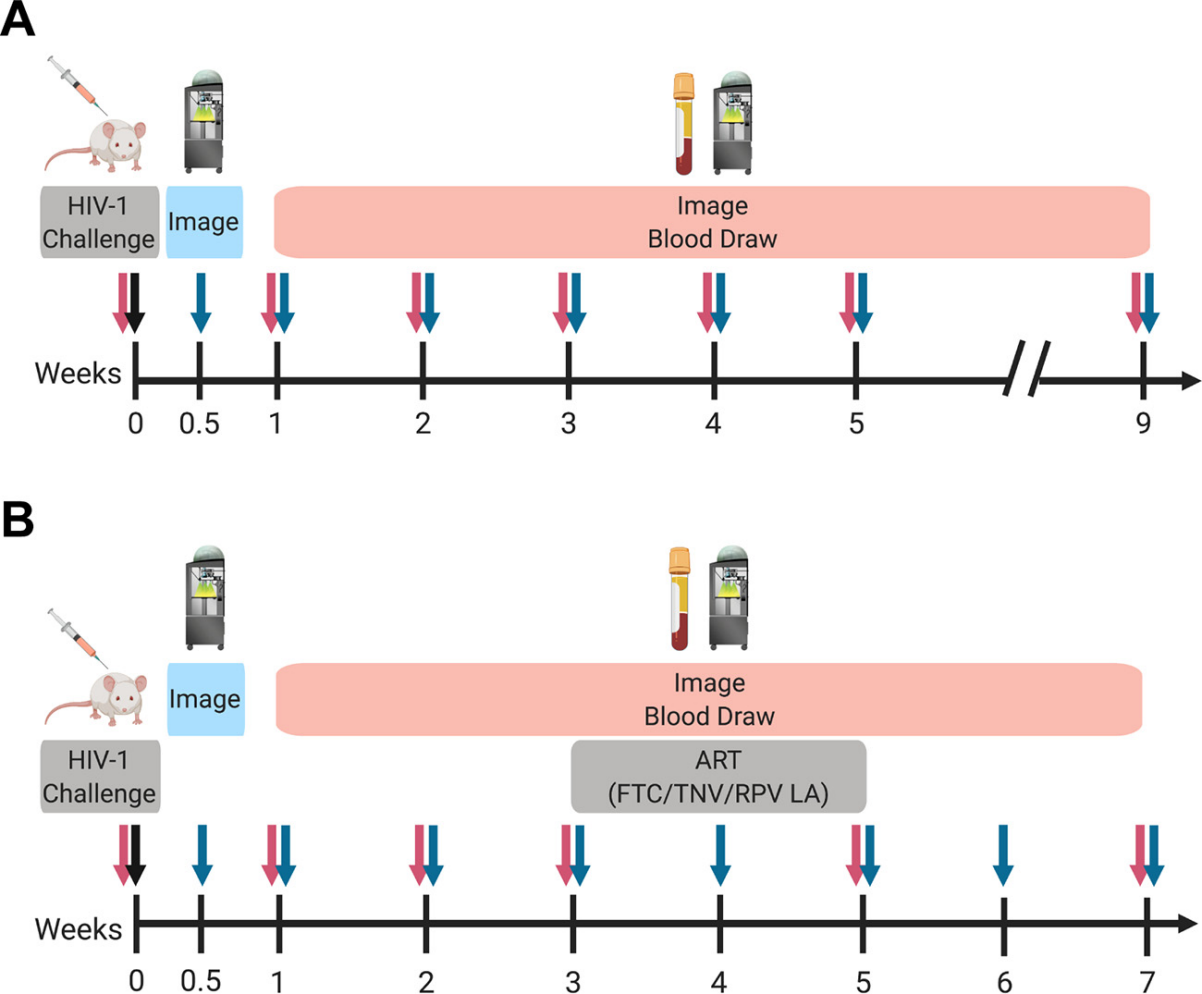
Design of experiments to characterize replication-competent HIV-1 reporter viruses in HIS mice. Original (A) or codon-optimized (B) nLuc HIV-1 or iRFP HIV-1 was injected i.p. into HIS mice. Virus replication was measured by qRT-PCR of viral RNA in plasma (red arrows) or by *in vivo* imaging (blue arrows). Daily ART was administered during weeks 3 to 5 postchallenge of codon-optimized viruses. Schematics were created with BioRender.com.

**FIG 3:**
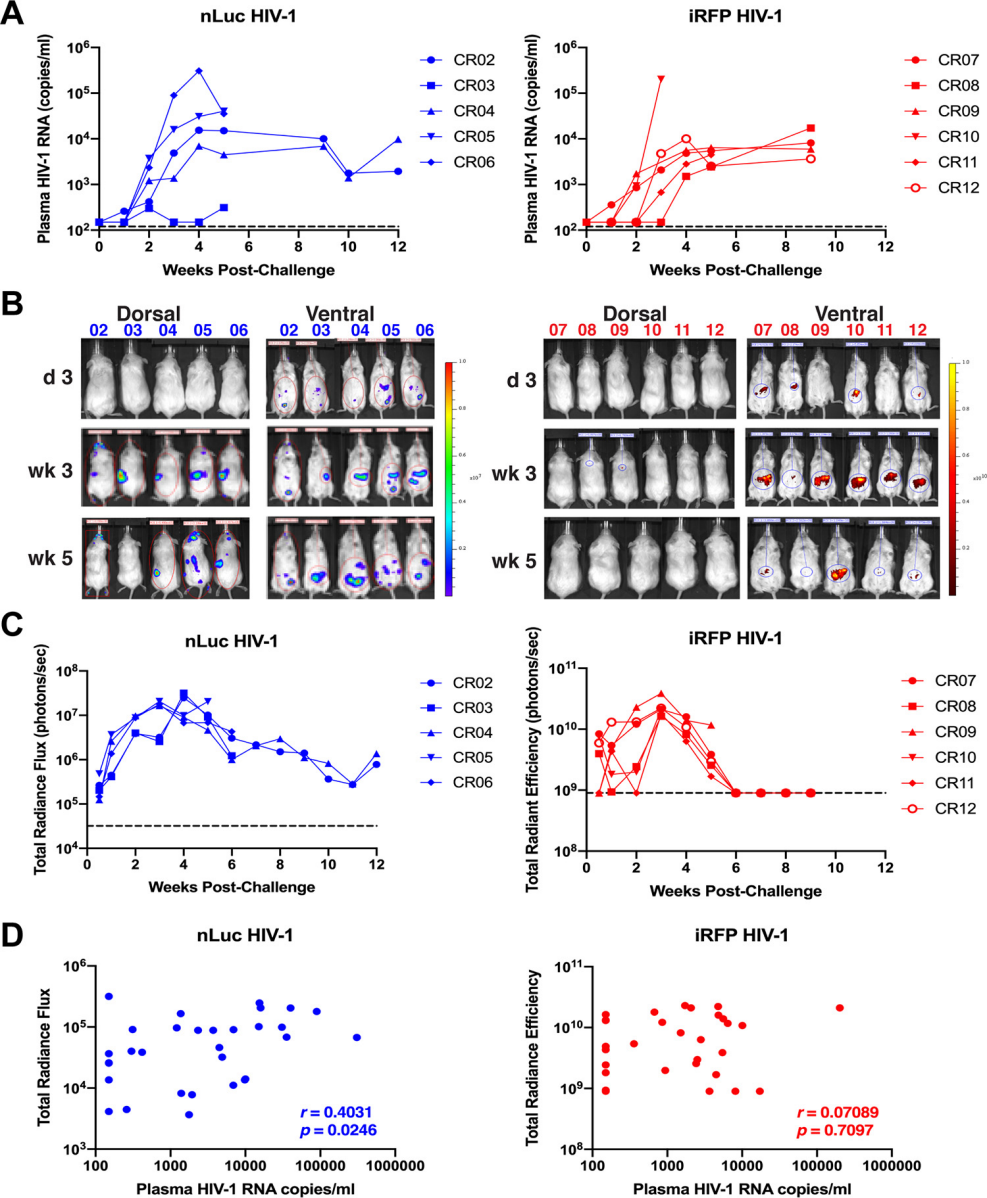
Low correlation of plasma viremia with *in vivo* imaging of original HIV-1 reporter viruses. HIS mice were challenged with nLuc HIV-1 (*n* = 5) or iRFP HIV-1 (*n* = 6). (A) Plasma viremia was measured by qRT-PCR. The limit of detection (120 HIV-1 RNA copies/ml) is denoted by the dashed lines. (B) Images of *in vivo* nLuc or iRFP expression measured by whole-body imaging are shown. (C) Expression of *in vivo* nLuc and iRFP expression was quantified at each time point. The limit of detection above background signal for the total radiance flux was 3.2 × 10^4^ photons/s, and that for the total radiant efficiency was 9 × 10^8^ photons/s (dashed lines). (D) Correlations of reporter gene expression with plasma viremia for all time points are shown. Spearman’s rank correlation coefficient (*r*) and *P* values are shown.

To determine reporter virus replication *in vivo*, mice engrafted with human CD34^+^ hematopoietic stem cells were injected intraperitoneally (i.p.) with 10^4^ infectious units (IU) of either nLuc HIV-1 (*n* = 5) or iRFP HIV-1 (*n* = 6; Fig. 2A). HIV-1 RNA was quantified weekly in the plasma of the mice for 5 weeks postchallenge by quantitative RT-PCR. A subset of mice were monitored until 9 to 12 weeks postchallenge. nLuc HIV-1 led to productive infection of four of five mice and iRFP HIV-1 infected all six mice, as measured by plasma viral RNA (Fig. 3A). Plasma viremia plateaued at week 4 postinfection in the majority of the mice and was maintained stably through at least 9 weeks postinfection, which was similar to our previous results using wild-type HIV-1_NL4-BAL_ infection of BLT mice (11).

Whole-animal imaging also was performed on the mice prior to infection, 3 and 7 days postinfection, and weekly thereafter. Bioluminescence imaging of nLuc HIV-1 in mice showed an exponential increase in signal between day 3 until week 3 or 4 postchallenge, including the one mouse with low to undetectable viremia (Fig. 3B and C). Mouse CR03 did not appear to be productively infected based on plasma viremia (Fig. 3A), yet nLuc expression was high, suggesting that CD4^+^ cells had been transduced but were not producing virus. However, nLuc expression decreased thereafter in the mice. *In vivo* fluorescence imaging of iRFP HIV-1 showed a smaller dynamic range compared to nLuc imaging, and iRFP expression peaked at week 3 postchallenge in all of the mice and decreased thereafter to undetectable levels (Fig. 3B and C) despite having relatively high viremia (Fig. 3A). While *in vivo* nLuc expression was generally correlated with plasma viremia (*P* = 0.025), *in vivo* iRFP expression did not correlate well with plasma viremia (*P* = 0.71), particularly for time points after week 4 or 5 postinfection (Fig. 3D). These data suggest that the reporter viruses replicated *in vivo*, but reporter gene expression diminished over time.

### Removal of CG dinucleotides in reporter genes improved reporter HIV-1 replication in vitro.

**FIG 4:**
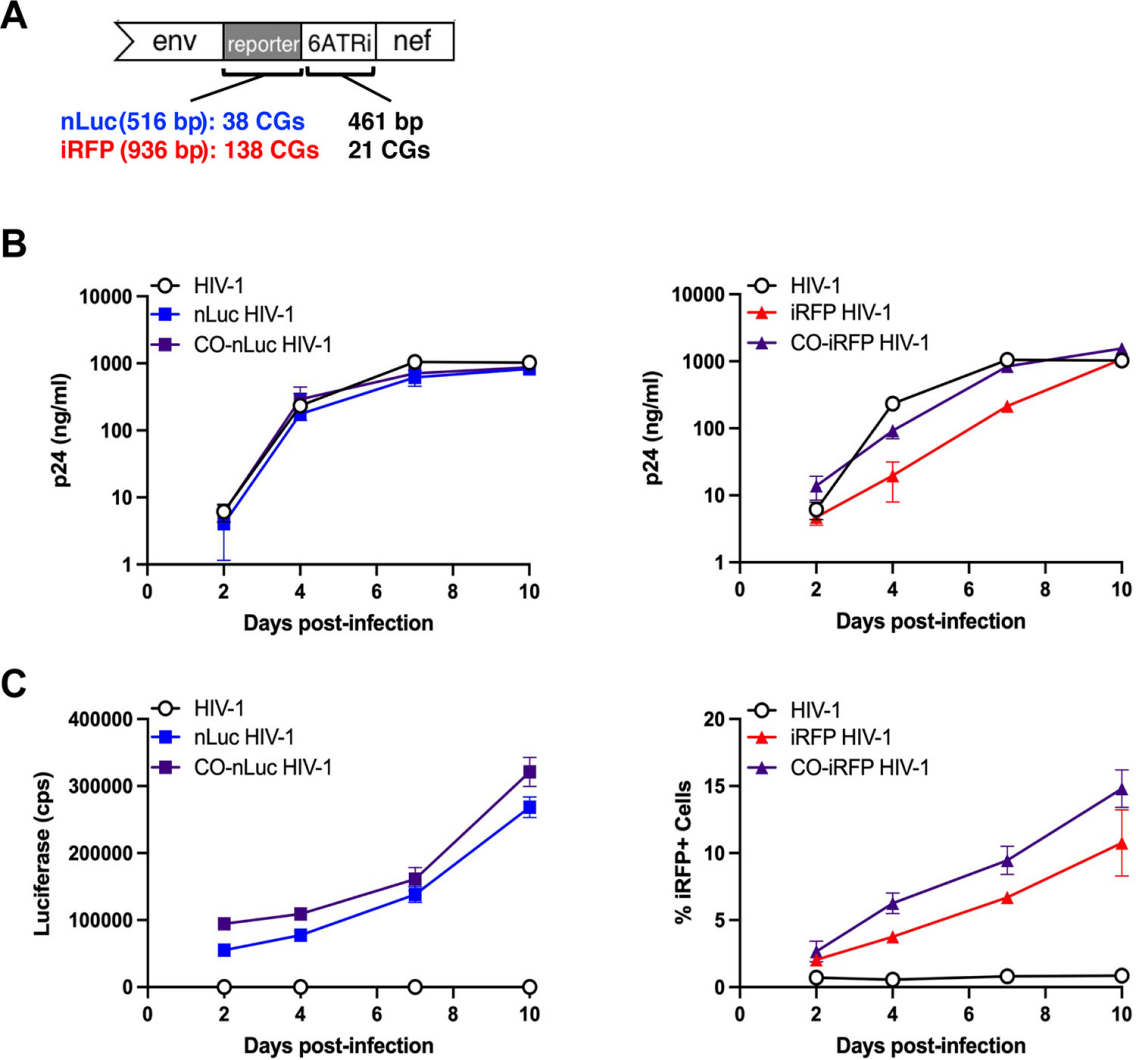
Synonymous mutations to abolish CG dinucleotides improves *in vitro* replication of HIV-1 reporter viruses. (A) CG dinucleotide sequences in the reporter genes (38 in nLuc and 138 in iRFP) were replaced by synonymous mutations. The 21 CGs in the 6ATRi sequence were not replaced. (B) PHA-stimulated primary PBMC were infected with wild-type HIV-1, original reporter viruses, or codon-optimized (CO) reporter viruses. Replication was measured by p24 production in the cell supernatant over time. The results are the average of three donors ± the SD. (C) nLuc (left) or iRFP (right) expression was measured in 20,000 PBMC (two donors) at each time point from cultures shown in panel B.

High frequencies of CG dinucleotides in HIV-1 have been shown to be associated with reduced virus replication due to recognition by the human zinc finger antiviral protein (ZAP) (12). We hypothesized that CG dinucleotides present in the nLuc and iRFP genes could lead to reduced virus replication and reporter gene expression. While the HIV-1 *gag* and *pol* genes (4,509 bp) had a total of 26 CG dinucleotides, nLuc (516 bp) and iRFP (936 bp) had 38 and 138 CGs, respectively (Fig. 4A). Synonymous base substitutions were introduced into the reporter genes of nLuc HIV-1 and iRFP HIV-1 to remove all CGs (see Fig. S1 in the supplemental material). Although the 6ATRi IRES (461 bp) had 21 CG dinucleotides, this sequence was not altered to avoid potentially affecting Nef expression.

Replication of codon-optimized (CO) reporter viruses was evaluated in peripheral blood mononuclear cells (PBMC) from three donors and compared to the original reporter viruses and wild-type (WT) HIV-1. Replication of both the original and the CO-nLuc viruses were indistinguishable from WT HIV-1 (Fig. 4B). While the original iRFP HIV-1 had lower replication compared to WT HIV-1, CO-iRFP HIV-1 replication was similar to that of WT HIV-1 (Fig. 4B). The kinetics of nLuc or iRFP expression in the PBMC showed that the reporters had slightly lower expression *in vitro* (Fig. 4C). These data suggest that fewer CG dinucleotides in the reporter genes resulted in improved reporter expression for both viruses and improved replication for iRFP-HIV-1 *in vitro*.

### Removal of CG dinucleotides in reporter genes improved reporter HIV-1 expression in vivo.

**FIG 5:**
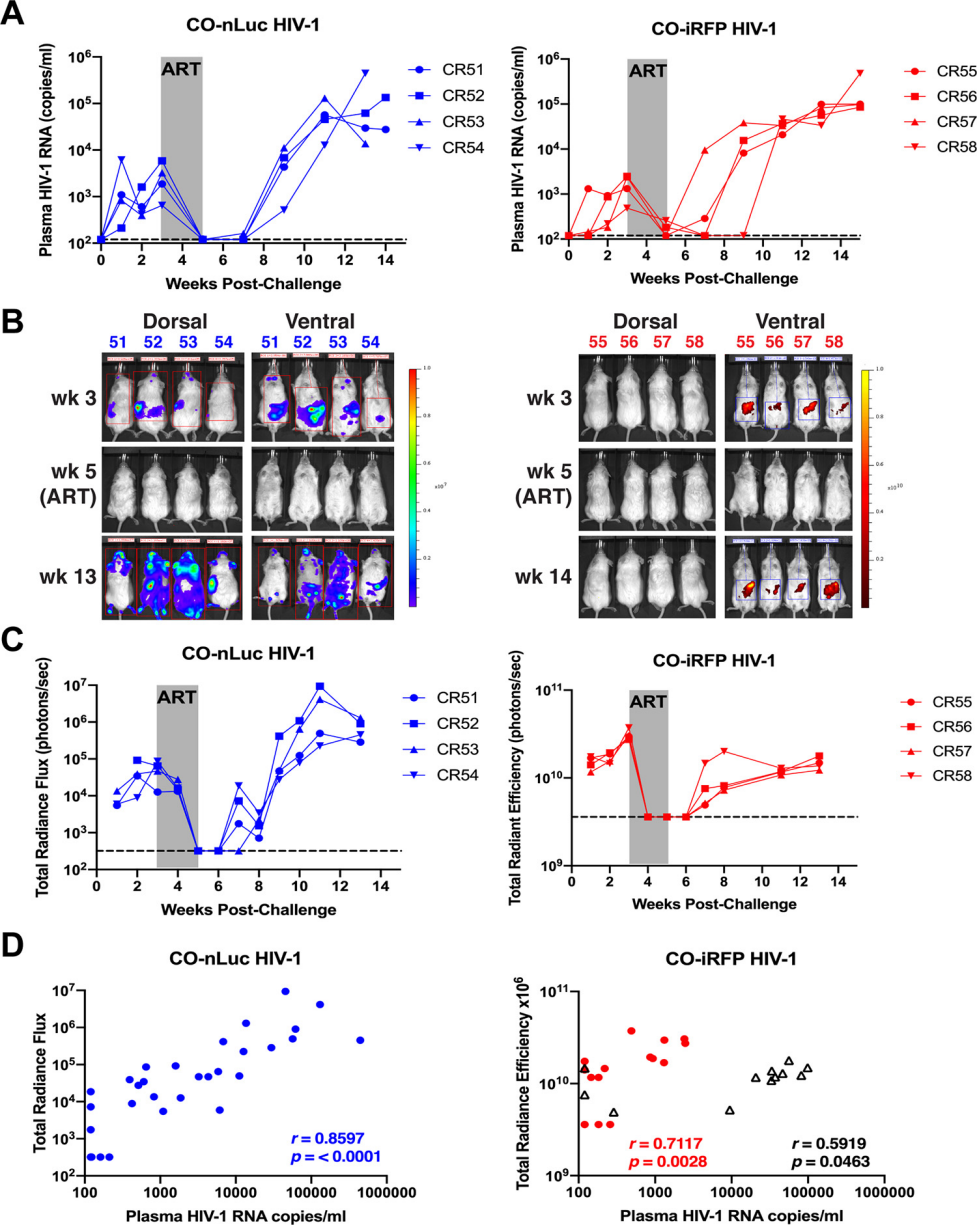
Codon optimization improves *in vivo* replication of HIV-1 reporter viruses. HIS mice were challenged with CO-nLuc HIV-1 (*n* = 4) or CO-iRFP HIV-1 (*n* = 4). (A) Plasma viremia was measured by qRT-PCR. The limit of detection (120 HIV-1 RNA copies/ml) is denoted by the dashed lines. Gray shading denotes period of ART administration. (B) Images of *in vivo* nLuc or iRFP expression measured by whole-body imaging are shown. (C) Expression of *in vivo* nLuc and iRFP expression was quantified at each time point. The limit of detection above background signal for the total radiance flux was 320 photons/s, and that for the total radiant efficiency was 3.9 × 10^9^ photons/s (dashed lines). Gray shading denotes period of ART administration. (D) Correlation of reporter gene expression with plasma viremia for all time points are shown. For CO-iRFP HIV-1, data points from weeks 1 to 5 (red circles) or weeks 7 to 14 (black triangles) are separated. Spearman’s rank correlation coefficient (*r*) and *P* values are shown.

Replication and reporter gene expression of the CO reporter viruses were evaluated in HIS mice (*n* = 4 per virus) for up to 15 weeks postchallenge (Fig. 2B). In addition, antiretroviral therapy (ART), consisting of daily tenofovir (TNV) and emtracitabine (FTC) and weekly long-acting rilpivirine (RPV LA), was administered to the mice between 3 and 5 weeks postchallenge to suppress virus replication. All animals that were injected with CO-nLuc HIV-1 or CO-iRFP HIV-1 became viremic prior to ART administration (Fig. 5A). Plasma viremia was reduced to undetectable or near undetectable levels in all mice during ART. As expected, viremia rebounded in all animals after ART cessation. Viremia rebounded to levels higher than pre-ART, suggesting that reservoirs may not have been completely established at the time of ART administration.

*In vivo* nLuc and iRFP expression was observed in all animals challenged with CO viruses prior to ART and declined to undetectable levels during ART, mimicking the trends seen for plasma viremia (Fig. 5B and C). Similar to the original iRFP HIV-1 animals, the dynamic range for *in vivo* detection was narrow compared to nLuc. After ART cessation, *in vivo* reporter virus detection increased or remained stable for at least 14 weeks. Reporter expression in animals infected with CO-nLuc HIV-1 correlated more strongly with plasma viremia levels (*P* < 0.0001; Fig. 5D) compared to the original reporter virus. Interestingly, the overall correlation between reporter expression and plasma viremia for mice infected with CO-iRFP HIV-1 was not significant (*P* = 0.2). However, if the data points were separated for prior to and during ART (weeks 1 to 5) or after ART administration (weeks 7 to 14), the correlation was significant for each data set (*P* = 0.0028 and *P* = 0.046, respectively).

**FIG 6:**
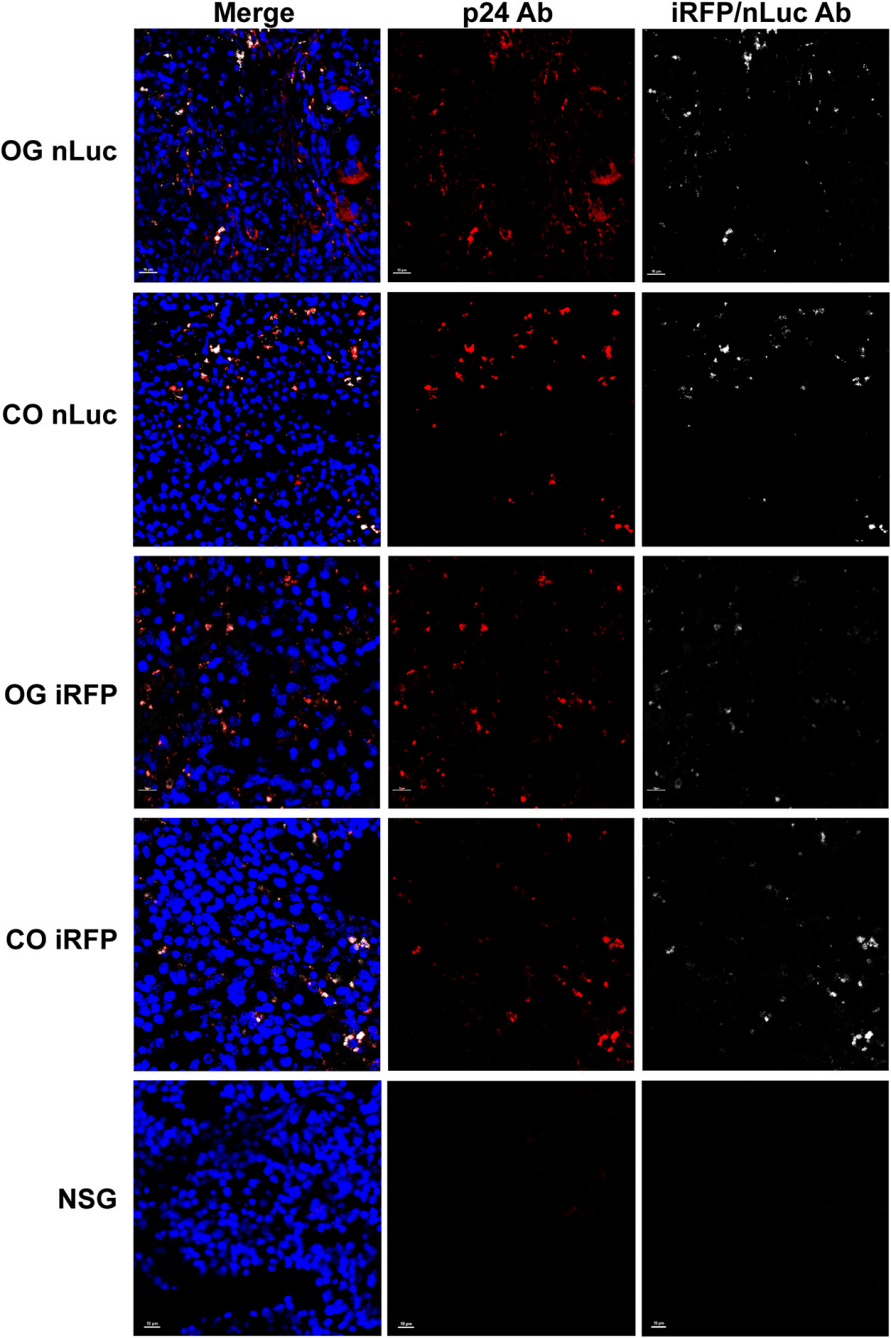
Reporter gene expression can be detected in HIV-infected cells in the spleens of HIS mice. Representative immunofluorescence images of Hoechst (blue), p24 (red), and reporter gene (white) expression in spleen sections of HIS mice infected with original (OG) nLuc HIV-1, CO-nLuc HIV-1, OG iRFP HIV-1, or CO-iRFP HIV-1. A NSG mouse lacking human cells was used as a negative control.

To evaluate expression of the reporters in the original and CO viruses at the cellular level, immunofluorescence was performed on spleens from the animals with productive infection. As animal CR03 did not have appreciable viremia, it was removed from analysis. Antibodies against HIV-1 capsid (p24) were used to stain sections of spleen from each animal (Fig. 6). For animals infected with the nLuc HIV-1 reporter viruses, staining also was performed using antibodies against nLuc.

**FIG 7:**
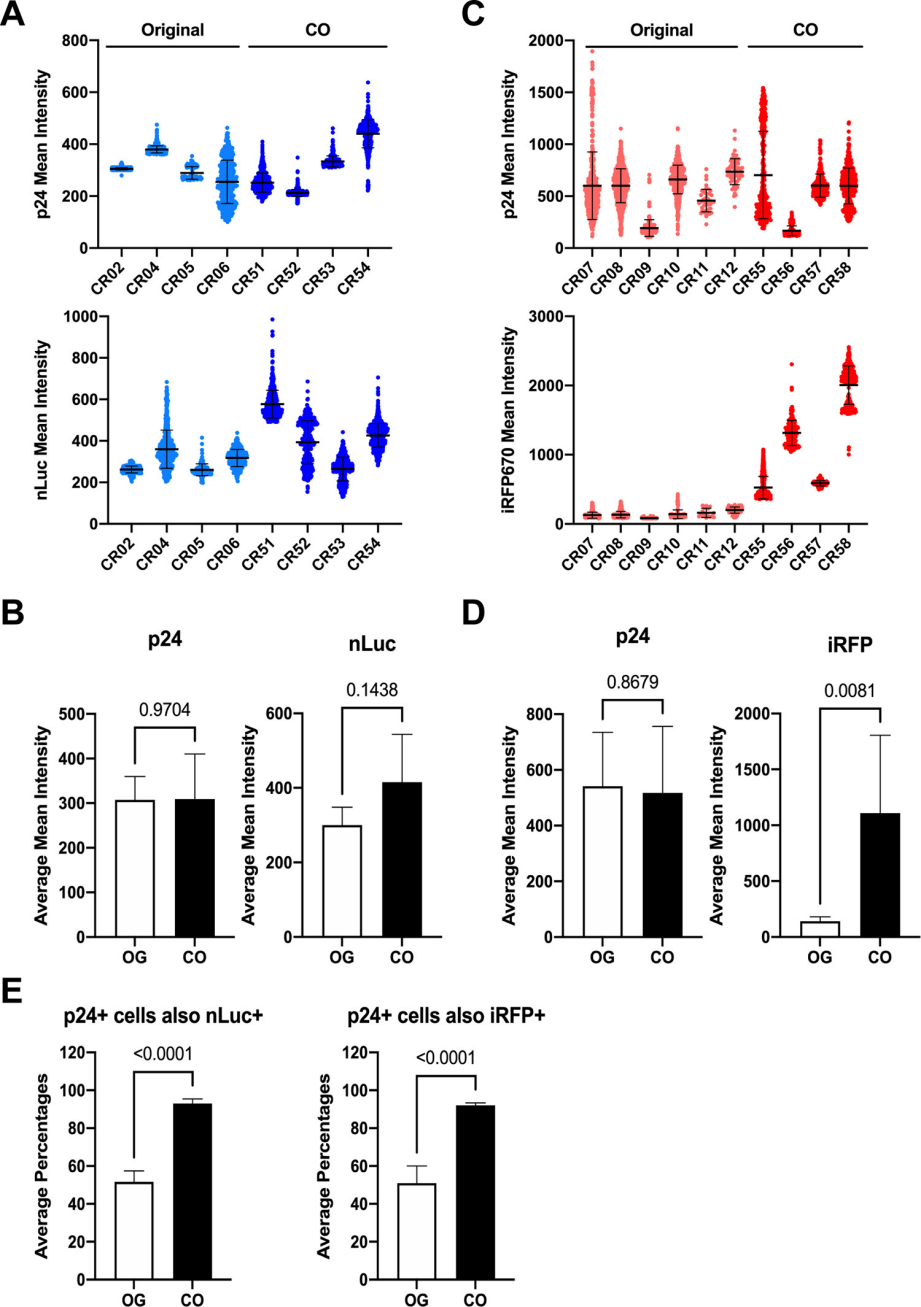
Expression of reporter proteins is improved in spleens of HIS mice infected with codon-optimized HIV-1 reporter viruses. Mean intensities of p24 and reporter gene expression in spleen cells of mice infected with nLuc/CO-nLuc HIV-1 (A) or iRFP/CO-iRFP HIV-1 (C) were measured by immunofluorescence imaging. The average number of positive cells analyzed per animal was 82 to 782 in four random fields of view. The average mean intensities of p24 and reporter expression in the nLuc/CO-nLuc HIV-1 (B) or iRFP/CO-iRFP HIV-1 (D) groups are shown. (E) The frequencies of p24^+^ cells that were also positive for nLuc or iRFP were counted for each animal. The means and standard deviations of each animal or group are shown. The *P* values from *t* tests are shown for all bar graphs.

First, expression intensity of the reporters was assessed and compared to p24 staining intensity. The intensity of spleen p24 staining showed variation between individual cells within a sample and between animals infected with the nLuc viruses (Fig. 7A), but the average mean intensities were nearly identical between the original and CO nLuc HIV-1 groups (*P* = 0.97; Fig. 7A and B). The average intensity of spleen nLuc signal in the animals infected with CO-nLuc HIV-1 was somewhat higher than that in the animals infected with the original reporter virus, but this was not statistically significant (*P* = 0.14; Fig. 7A and B). The p24 staining intensity in the spleens of animals infected with the original iRFP HIV-1 was very heterogeneous (Fig. 7C) and higher than the animals infected with original nLuc HIV-1 (*P* = 0.049). However, the difference in average mean intensities between animals infected with the original or the CO nLuc HIV-1 was similar (*P* = 0.87; Fig. 7C and D). In contrast to nLuc expression, iRFP could be visualized without antibodies or substrate. The mean signal intensity was significantly higher in animals infected with CO-iRFP HIV-1 than those infected with the original virus (*P* = 0.0081; Fig. 7C and D).

Since immunofluorescence imaging appeared to show some p24^+^ cells that were not positive for reporter gene expression in animals that were infected with the original reporter viruses (Fig. 6), we evaluated the number of p24^+^ cells that were also positive for reporter expression for all animals. While the frequency of double-positive infected cells was on average approximately 50% for animals infected with the original nLuc or iRFP viruses, >90% of p24^+^ cells also had detectable reporter gene expression in animals infected with the CO reporter viruses (Fig. 7E). Overall, the *in vivo* and *ex vivo* data suggest that CG codon optimization of the reporter genes significantly improved their expression.

### Deletion of reporter and IRES sequences of viral RNA occurs in animals infected with reporter viruses.

**FIG 8:**
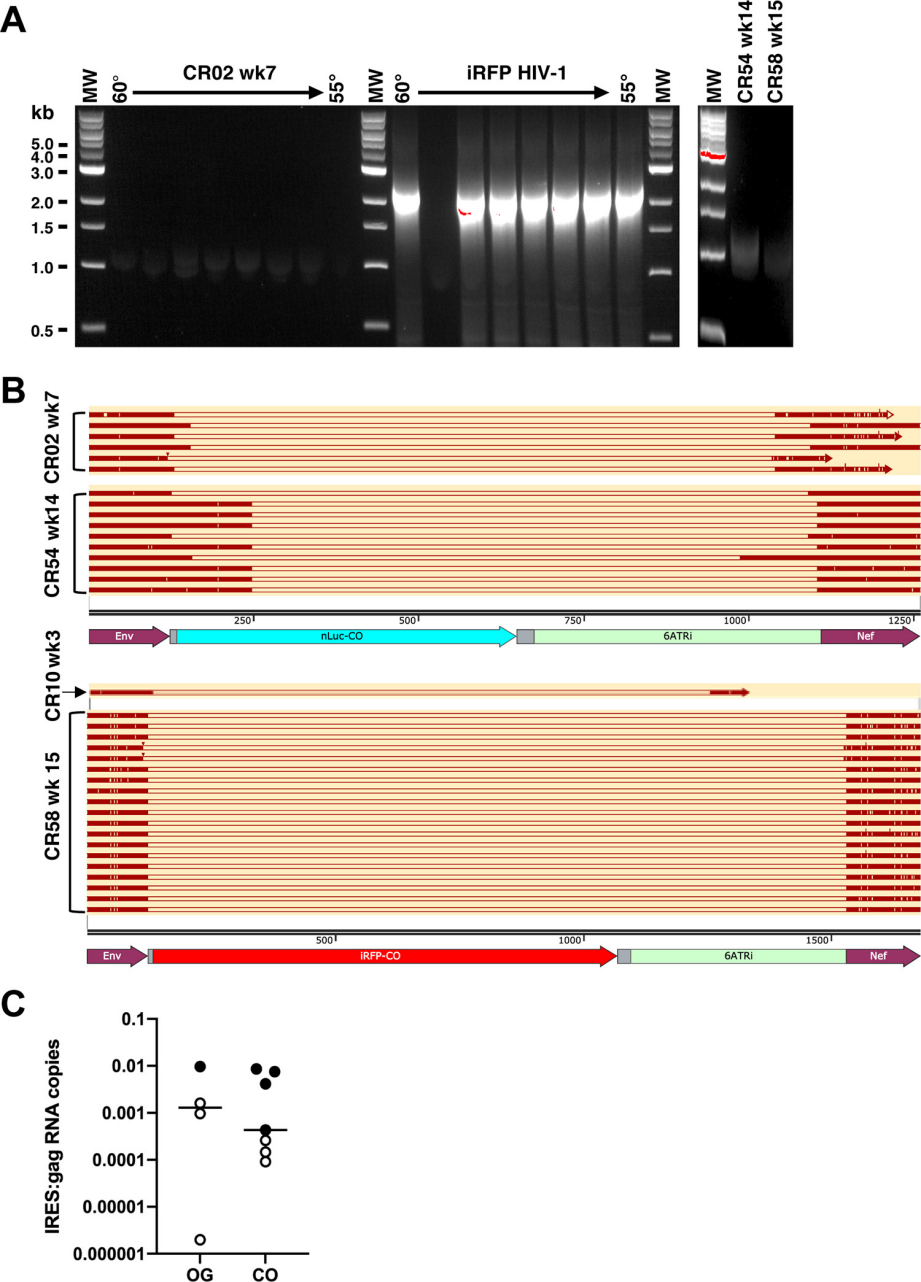
Reporter gene and IRES sequences are deleted in plasma HIV-1 RNA. (A) Representative agarose gels of RT-PCR products amplified from the reporter and 6ATRi region from HIV-1 RNA isolated from challenge iRFP HIV-1 stock and plasma from animals CR02 (week 7), CR54 (week 14), and CR58 (week 15). The challenge stock amplified a band of the expected size (2,452 bp), while the only detectable band was smaller than expected and cloned into a plasmid vector. (B) Multiple plasmid clones were sequenced. Plasma sequences that aligned with the proviral molecular clones are shown in dark red bars and missing sequences are shown open bars. (C) The ratios of IRES RNA copies to *gag* RNA copies quantified by qRT-PCR from plasma HIV-1 RNA are shown for individual animals infected with OG or CO viruses that had >10^3^ gag copy numbers/reaction. Closed symbols indicate that >9 copies of 6ATRi sequences were detected in a sample, and open symbols indicate no detectable 6ATRi sequences were detected.

While plasma viral RNA and intracellular p24 expression were not impaired in the original reporter viruses, reporter expression was significantly decreased, particularly for iRFP HIV-1. The fact that reporter gene expression is derived from the 5′ LTR, as are all other viral proteins, suggests that reporter gene sequences may have been mutated or deleted. Viral RNA was extracted from plasma from animals infected with the original nLuc HIV-1 (CR02, week 7), CO-nLuc HIV-1 (CR54, week 14), original iRFP HIV-1 (CR10, week 3), or CO-iRFP HIV-1 (CR58, week 15) and converted to cDNA. Primers in *env* and *nef* were used to amplify the region containing the reporter gene and IRES. However, only bands at sizes consistent with loss of the reporter gene and IRES were detectable on an agarose gel. Sequencing of several clones from these four mice revealed that in all sequences, most of the reporter gene and the IRES were deleted (Fig. 8A and B).

To quantify the frequency of plasma RNA copies containing the IRES sequence, and thus likely the reporter gene, quantitative RT-PCR was performed on plasma samples using primers and probe at the 5′ end of the 6ATRi IRES region, which is identical for all four viruses and was missing in amplified sequences. The copy numbers containing the IRES were significantly lower than for *gag*. Since many animals had low *gag* copy numbers per PCR due to relatively small amounts of viral RNA and low volumes of plasma remaining after viremia measurements, only a subset of animals were evaluated that had at least 10^3^ *gag* copies. IRES sequences were undetectable (<9 copies/reaction) in three of four mice infected with original reporter viruses, with the one detectable sample coming from animal CR10 that was euthanized early (week 3 postinfection), and four of seven mice infected with CO reporter viruses (Fig. 8C). While the ratios of IRES to gag copies were not significantly different between the groups, the data suggest that deletion of the reporter gene sequence can occur *in vivo*, which may result in loss of nLuc or iRFP expression in cells.

### Dual bioluminescence and fluorescent imaging of two HIV-1 variants in vivo.

**FIG 9:**
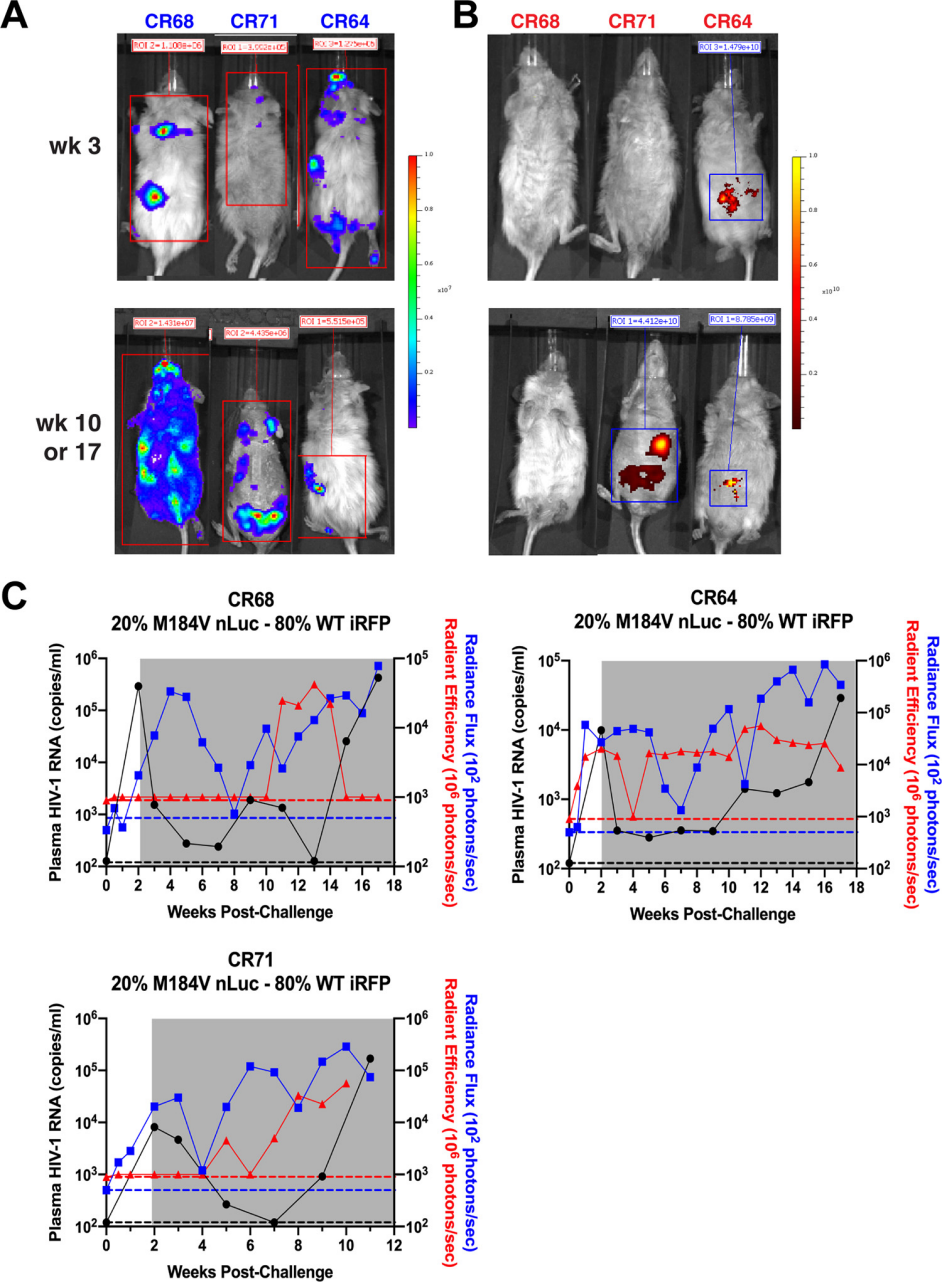
Dual imaging of CO-nLuc HIV-1 and CO-iRFP HIV-1 can be performed in mice. Representative whole-body images of three HIS mice infected with 20% CO-nLuc HIV-1 with the M184V RT mutation and 80% CO-iRFP HIV-1 are shown for nLuc (dorsal side) (A) and iRFP (ventral side) (B). ART was initiated at week 2 postinfection through the end of the study. Week 3 results are shown for all animals, week 10 results are shown for CR71, and week 17 results are shown for CR64 and CR68. (C) Plasma viremia, total radiance flux for nLuc, and total radiant efficiency for iRFP over time are shown for each animal. Gray shading indicates daily ART and dashed lines indicate the limit of detection for viremia (black), nLuc (blue), and iRFP (red).

To determine whether dual imaging could be performed to visualize both CO-nLuc HIV-1 and CO-iRFP HIV-1 in the same animals, mice engrafted with human CD34^+^ hematopoietic stem cells and autologous fetal thymus, liver, and spleen tissues were infected with a virus population consisting of 80% CO-iRFP HIV-1 and 20% CO-nLuc HIV-1 containing the M184V amino acid substitution in reverse transcriptase (RT). RT M184V confers high-level resistance to FTC. ART that included FTC was initiated at week 2 postinfection through the last time point (week 11 for animals CR71 or week 17 for animals CR64 and CR68). Both nLuc and iRFP signal could be detected at multiple time points in all animals (Fig. 9). ART reduced HIV-1 replication by >1 log in all animals, but over time plasma viremia increased to pre-ART levels or higher (Fig. 9C). Rebound plasma viremia was not associated with high frequencies of additional drug resistance mutations in RT (data not shown). Expression of nLuc remained high for several weeks after ART administration, suggesting that reporter gene expression in tissues could remain high early during ART in the absence of virus replication. Detection of iRFP was high for one animal (CR64) and remained low in the other animals until later time points, which was associated with an increase in nLuc expression that preceded detection of plasma viremia. These results suggest that both reporter viruses can be detected in coinfected animals and that gene expression in tissues precedes detection of plasma viremia in HIS mice. Future studies will be needed to understand the reservoir dynamics associated with virologic failure in these animals.

## DISCUSSION

In this study, we evaluated HIV-1 infection over time by whole-body imaging in HIS mice using replication-competent molecular clones encoding either a bioluminescent protein (nLuc) or a near-infrared fluorescent protein (iRFP). Both reporter viruses were detectable in HIS mice infected with a single virus or with both viruses. Each reporter has advantages and disadvantages in *in vivo* imaging. While nLuc is almost half the size of a fluorescent protein (19 and 34.5 kDa, respectively) and emits a strong signal with little background, it requires a substrate (furimazine) for detection of luminescence. Use of iRFP is attractive because it does not require administration of a substrate to the animals, allowing easier visualization of infected cells by immunohistochemistry and the ability to isolate infected cells postmortem, which is particularly important for relatively small populations of infected cells or in latency studies. Despite being in the optimal window for deep tissue imaging (13, 14), iRFP had higher background and a narrower dynamic range of detection above background compared to nLuc. However, reporter gene expression in live HIS mice decreased after ∼1 month postinfection and was not well correlated with plasma viremia, particularly for iRFP HIV-1.

Removal of CG dinucleotides increased virus replication levels of both viruses *in vitro*. The iRFP gene is twice the size of the nLuc gene but had more than 3.5-fold the number of CG dinucleotides, which was consistent with greater defects in reporter virus replication and *in vivo* gene expression prior to codon optimization. While replication of the original and CO reporter viruses were not directly compared in mice made from the same donors, which could limit variability in reconstitution of the mice and virus replication, it was clear that removal of CGs significantly improved reporter gene expression *in vivo*. Both the *in vivo* and *ex vivo* expression intensity as well as the correlation of reporter expression in p24^+^ cells were improved for both reporter viruses after correction of CG dinucleotides. *In vivo* fluorescent reporter signal possibly could be improved by using a more red-shifted fluorescent protein, such as iRFP720 (14, 15), and correction of CGs in the IRES.

In addition to sustained reporter expression, both CO reporter viruses were suppressed during ART. Surprisingly, viremia increased above pre-ART levels following discontinuation of short-term ART in the HIS mice reconstituted with CD34^+^ cells. Higher than expected rebound is likely due to a viral set point, and thus reservoirs, not having been completely established at the time of ART initiation. Data from the original reporter viruses suggest that the plasma viral set point was not achieved until week 4 postinfection. It is also possible that immune reconstitution was continuing in the mice, which would alter reservoirs. HIS mice engrafted with CD34^+^ cells and liver/thymus were used for the dual imaging study and likely have better tissue reconstitution with human cells, particularly for myeloid cells. In this study, reporter gene expression remained relatively high during ART, including in the absence of virus replication, suggesting that gene expression can continue after suppression of HIV-1 replication. Similarly, gene expression preceded detectable viremia when therapy failed and virus rebounded.

Multiple factors could explain differences in reporter gene expression compared to detectable plasma viremia. First, it has been shown that the majority of HIV-1 proviruses are defective over the course of infection (16, 17). Defective proviruses that do not produce virus can still express viral proteins (18, 19). Indeed, one animal was never productively infected (i.e., no detectable plasma viremia), despite high detectable nLuc expression *in vivo*. It is possible that reporter expression occurred from defective proviruses that were unable to produce infectious virions. Intact proviruses that are capable of producing replication-competent viruses are associated with a deeper state of transcriptional latency, requiring stronger and/or longer cellular activation for expression compared to cells with defective proviruses (20). Over time, CD4^+^ T cells harboring intact proviruses can decay more rapidly over time than cells containing defective proviruses (21). This may be the case for the animals infected with CO-iRFP HIV-1, which has a relatively large inserted sequence in the genome, that had lower reporter gene expression (even with codon optimization) following short-term ART cessation.

HIV-1 reservoirs and virus production also can be affected by clonal expansion of individual cells (22, 23). Decay of HIV-infected cells during ART are competing with clonal expansion of other cells (24). Indeed, a recent study shows that ART-treated individuals can have nonsuppressive viremia in the absence of high-level drug resistance due large frequencies of clonally expanded cells containing wild-type HIV-1 (25). This could explain virologic failure in our animals treated with 10 or more weeks of suppressive ART. Further studies will be needed to determine decay rates and clonal expansion of human HIV-infected cell subsets in HIS mice and whether their kinetics and population sizes are representative of those measured in humans.

The codon-optimized reporter viruses we describe here can be used to study replication of two viral variants *in vivo*. This may be particularly useful in development of new treatment strategies, including those for latency reversal or induction. Increase in reporter expression during ART can be predictive of treatment failure or reversal of latency, which can be assessed more rapidly and possibly more sensitively than quantitative RT-PCR assays. In addition, location of virus replication in particular cells or tissues can be easily determined.

## MATERIALS AND METHODS

### Cells.

HEK293T cells were cultured in DM-10 medium: Dulbecco modified Eagle medium (Thermo Fisher) supplemented with 10% fetal bovine serum (FBS; Atlanta Biologicals) and 100 U/ml of penicillin, 100 μg/ml of streptomycin, and 0.292 mg/ml of l-glutamine (PSG; Thermo Fisher). GHOST-R3/X4/R5 cells were maintained in DM-10 containing 100 μg/ml G418 (Thermo Fisher Scientific), 100 μg/ml hygromycin (Thermo Fisher Scientific), and 0.5 μg/ml puromycin (EMD Millipore).

HuT-R5 cells were cultured in RP-10 medium: RPMI (Thermo Fisher) supplemented with 10% FBS and PSG. PBMC were isolated from blood obtained from an HIV-1 uninfected donors at the Central Blood Bank overlaid on lymphocyte separation medium (MP Biomedicals). Cells were stimulated for 48h with 5 μg/ml phytohemagglutinin (PHA) in RP-10 containing 50 U/ml interleukin-2 (IL-2; Roche).

### Viruses.

A proviral plasmid encoding HIV-1_NL4-BAL_ (26) was modified to encode nLuc or iRFP upstream of an IRES (6ATRi) between *env* and *nef* in the same manner previously described (8) using gene synthesis (GenScript). Subsequently, the nLuc and iRFP genes were resynthesized to remove all CG dinucleotides with synonymous mutations. These proviral constructs and their full sequences will be made available at the NIH HIV Reagent Program. The M184V amino acid substitution in RT was introduced into CO-nLuc HIV-1 using site-directed PCR mutagenesis (QuikChange kit; Agilent). The Rous sarcoma virus retroviral vector system (RCAS) (27) was used as an internal control for plasma viremia.

Viruses were produced by transfection of HEK293T cells with proviral plasmids using Lipofectamine 2000 (Thermo Fisher). Cell culture supernatant containing HIV-1 was filtered through a 0.45-μm filter, concentrated with Lenti-X (TaKaRa), and frozen in aliquots at −80°C. Virus titers were determined in duplicate on GHOST cells (9) by limiting dilution and flow cytometry on a BD Accuri flow cytometer (BD Biosciences).

### Antiretroviral drugs.

RPV LA (300 mg/ml) was obtained from Janssen R&D Ireland and stored at 4°C until use. TNV and FTC were obtained from Gilead Sciences and formulated at 60 mg/ml and 110 mg/ml, respectively, in distilled water (pH 7.0).

### Infection assays.

Replication assays were performed in HuT-R5 cells or PHA-stimulated PBMC. Cells were incubated with HIV-1 at an MOI of 0.01 (HuT-R5) or 0.05 (PBMC). Production of capsid (p24) antigen was measured by enzyme-linked immunosorbent assay (Xpress Bio) in supernatants taken between 2 and 10 days postinfection after filtration through a 0.45-μm syringe filter. Expression of nLuc in cells was determined after cell lysis of a specific number of cells by using the Nano-Glo luciferase assay system (Promega). Expression of iRFP in cells was determined after fixation by flow cytometry and reported as a percentage.

### Ethics statement.

All animal-related work was conducted according to the Public Health Services (http://grants.nih.gov/grants/olaw/references/PHSPolicyLabAnimals.pdf). Mice were housed at the University of Pittsburgh Division of Laboratory Animal Resources at the UPMC Hillman Cancer Center in accordance with the American Association of Accreditation of Laboratory Animal Care standards. All procedures were approved by the University of Pittsburgh Institutional Animal Care and Use Committee under protocol 17020145. Animals were housed in microisolator cages and monitored daily for behavior, appearance, and physiology. Mice had unlimited water, which was autoclaved, chlorinated, and treated on alternate weeks with sulfamethoxazole-trimethoprim, and dry food. In addition, DietGel 76A cups (ClearH2O) often were provided in cages as a supplementary diet postprocedure (i.e., blood collection). Procedures were conducted while the animals were sedated either with an intramuscular (i.m.) injection of ketamine (Henry Schein, 44 mg/kg) or inhalation of nebulized isoflurane mixed with oxygen (5% in 70% O_2_/30% NO_2_). Animals were euthanized at the endpoint of the study with 100% carbon dioxide at a flow rate of 20 to 30%. Any animal that failed to thrive (i.e., loss of 20% body weight from the start of the experiment), ambulate, perform normal mouse behavior, or obviously moribund was also euthanized.

### Infection of HIS mice.

NSG (NOD.*Cg-Prkdc^scid^Il2rg^tm1Wjl^*/SzJ) mice reconstituted with human CD34^+^ cells were purchased from Jackson Laboratory (Table 1). These mice were challenged i.p. with 10^4^ IU with either nLuc HIV-1 or iRFP HIV-1 at 17 to 21 weeks postengraftment (Table 1). Whole-body imaging was performed at days 3 and 7 postchallenge and weekly thereafter (see below). Approximately 40 to 75 μl of blood was collected via aseptic submandibular venipuncture with a lancet prior to and after virus challenge. Animals infected with CO viruses received ART consisting of daily TFV and FTC administered i.p. (150 and 275 mg/kg, respectively) and weekly RPV LA administered i.m. (150 mg/kg) between weeks 3 to 5 postchallenge.

**TABLE 1:** CD34^+^ HIS mouse information

Animal	Reporter virus	hCD45^+^ PBMC (% total)^*a*^	hCD3^+^ cells (% of hCD45^+^)^*a*^^,^^*b*^	hCD33^+^ cells (% of hCD45^+^)^*a*^^,^^*c*^	Challenge (wk postengraftment)	Necropsy (wk postchallenge)
CR02	nLuc-OG	29.8	41	9	21	12
CR03	nLuc-OG	32.1	56	6	21	6
CR04	nLuc-OG	34.6	40	5	21	12
CR05	nLuc-OG	35.2	43	8	21	5
CR06	nLuc-OG	30	37	12	21	6
CR07	iRFP-OG	32.6	29	15	21	9
CR08	iRFP-OG	36.3	40	12	21	9
CR09	iRFP-OG	36.2	39	10	21	9
CR10	iRFP-OG	41.1	29	11	21	3
CR11	iRFP-OG	40.7	41	9	21	6
CR12	iRFP-OG	42.5	48	9	21	9
CR51	nLuc-CO	77.7	21	3	17	14
CR52	nLuc-CO	69.6	23	4	17	14
CR53	nLuc-CO	81.6	18	4	17	13
CR54	nLuc-CO	63.2	32	1	17	14
CR55	iRFP-CO	54.5	32	6	17	15
CR56	iRFP-CO	59.4	15	6	17	15
CR57	iRFP-CO	64.6	17	3	17	15
CR58	iRFP-CO	52.1	11	3	17	15

a Measured at 8 weeks postengraftment.

b Human T lymphocytes

c Human myeloid cells.

NSG mice reconstituted with autologous human CD34^+^ cells and fetal liver, thymus, and spleen were purchased from the Human Immune System Mouse Program at Massachusetts General Hospital. These mice were challenged i.p. with 5 × 10^4^ IU (CR64) or 5 × 10^3^ IU (CR68 and CR71) consisting of 80% CO-iRFP HIV-1 and 20% CO-nLuc HIV-1 with the M184V mutation in RT. Beginning at week 2 postchallenge, ART was administered as described above until euthanasia, which was performed once virologic failure was confirmed by plasma viremia.

### Quantitative PCR.

Plasma HIV-1 RNA was isolated as previously described and quantified by SCA (11). Briefly, a known amount of RCAS was spiked into each plasma sample as an internal RNA isolation control, and HIV-1 and RCAS were pelleted by centrifugation at 4°C. Total viral RNA was isolated with guanidinium isothiocyanate and glycogen, and cDNA synthesis was performed with random hexamers. TaqMan quantitative PCR (qPCR) was performed in duplicate for all samples. HIV-1_NL4-BAL_ *gag* and RCAS RNA transcripts for standard dilutions were synthesized from plasmids encoding the region of amplification using the RiboMAX large-scale RNA production system (Promega). Data were only reported from samples in which RCAS was successfully amplified. The limit of quantitation of SCA from the plasma volumes was 120 HIV-1 RNA copies/ml plasma.

TaqMan quantitative RT-PCR for the 6ATRi sequence was performed using an RNA standard produced by *in vitro* transcription and the following primers: 5′-ATGCAAGGTCTGTTGAATGTCG-3′ (forward), 5′-ACTCACAACGTGGCACTGG-3′ (reverse), and 5′-R-CGTGTATAAGATACACCTGCAAAGGCG-Q-3′ (probe where R represents fluorescein dye and Q is the nonfluorescent quencher, minor groove binder).

### *In vivo* imaging.

For *in vivo* whole-body imaging for nLuc, mice were injected i.p. with 0.5 ml of NanoLuc luciferase substrate (Promega) diluted 1:100 in sterile phosphate-buffered saline (PBS). Mice infected with iRFP HIV-1 did not require administration of substrate and was used as a control for nLuc. Mice were anesthetized with nebulized isoflurane mixed with oxygen in an air-sealed chamber. Images were obtained with a Xenogen IVIS-200 (Perkin-Elmer) using luminescent filters for nLuc or Cy5.5 filters (both excitation and emission) for iRFP. All images were quantified for reporter expression using Living Image Software (Perkin-Elmer) according to the manufacturer’s instructions.

### Immunofluorescence.

Portions of spleen tissues were collected at necropsy (Table 1) from all viremia HIS mice infected with an original reporter virus (*n* = 5 to 6/group) or CO reporter viruses (*n* = 4/group). The spleens were fixed and cryopreserved at −80°C. Thin sections (10 μm) of spleen were cut with a microtome and kept at –20°C until they were ready for use. Tissues were rehydrated with two washes of PBS and then permeabilized with PBS containing 0.1% Triton X-100. Tissues were blocked with buffer containing 2% bovine serum albumin (BSA) and 20% donkey or goat serum for 45 min. Staining was performed with antibodies against HIV-1 p24 (1:1,500; Santa Cruz Biotechnology, clone 24-4) and nLuc (1:1,000; R&D Systems, clone 965808), followed by washing with PBS containing 0.5% BSA (PBB) and incubation with secondary antibody: donkey anti-mouse IgG-Cy3 (1:2,000; Jackson Immunological) or goat anti-mouse IgG-Cy5 (1:1,500; Abcam) After a washing step, Hoechst stain (Invitrogen) was added to stain the nuclei, and the sections were washed again with PBB. Coverslips were mounted using gelvetol.

Immunofluorescence of p24, nLuc, and iRFP was measured by confocal microscopy. A Nikon A1 spectral inverted confocal microscope with a 40 × 1.49 NS oil immersion objective (Nikon) was used to acquire images of the fixed tissue samples. LU-NV laser launch (Nikon) was used to emit lasers at 405 nm (Hoechst), 561 nm (Cy3), and 640 nm (Cy5/iRFP). For each tissue section, multiple images were acquired from different randomly chosen fields of view. NIS-Elements software (Nikon) was used to analyze the fluorescence intensity and frequency of fluorescent cells. To analyze *in vivo* HIV-1 reporter expression, the entire tissue area was segmented based on Hoechst signal to define each segment as a cell with nucleus. Then, for each respective channel (Cy3.5 for HIV-1 p24 and Cy5 for iRFP or nLuc), positive cells were determined. Cells having positive immunofluorescence were selected as binary zones, which were converted to regions of interest to measure mean fluorescence intensity of each positive cell with iRFP/nLuc and p24 signal. The intensities of the reporter and p24 expression levels in spleen sections were plotted for each mouse.

### HIV-1 sequencing.

PCRs were performed to amplify the 3′ end of *env* and the reporter gene (5′-AGCAGCTCCAGGCAAGAGTCC-3′) through the 5′ portion of *nef* (5′-TCCTCCTCTTGTGCTTCTAGCC-3′) of cDNA produced from 3 μl of the challenge virus stock and plasma RNA from a subset of mice in each group using RANGER Mix (Bioline). The PCR conditions were as follows: 95°C for 2 min; 35 cycles each of 95°C for 30 s, 60°C for 30 s, and 72°C for 4 min; and a final extension of 72°C for 5 min. PCR products were Topo cloned into the pCR4-TOPO TA vector (Thermo Fisher) and grown in TOP10 Escherichia coli (Thermo Fisher). Multiple plasmid clones were isolated and sequenced by Sanger sequencing (Genewiz). Alignments were performed using SnapGene, version 5.2.4.

### Statistical analyses.

Statistics were performed using Prism 9.0.1 (GraphPad). Spearman correlations were performed to compare *in vivo* imaging and plasma viremia in each animal group. The mean average intensities of reporter gene expression were plotted per group and compared using unpaired *t* tests.

## References

[1] Garcia-Lerma JG, Heneine W. 2012. Animal models of antiretroviral prophylaxis for HIV prevention. Curr Opin HIV AIDS 7:505–513. 10.1097/COH.0b013e328358e484. 22964889

[2] Del Prete GQ, Lifson JD, Keele BF. 2016. Nonhuman primate models for the evaluation of HIV-1 preventive vaccine strategies: model parameter considerations and consequences. Curr Opin HIV AIDS 11:546–554. 10.1097/COH.0000000000000311. 27559710 PMC5100008

[3] Ambrose Z, KewalRamani VN. 2008. Of mice and monkeys: new advances in animal models to study HIV-1 therapy and prophylaxis. Future HIV Ther 2:363–373. 10.2217/17469600.2.4.363.

[4] Whitney JB, Brad Jones R. 2018. *In vitro* and *in vivo* models of HIV latency. Adv Exp Med Biol 1075:241–263. 10.1007/978-981-13-0484-2_10. 30030796

[5] Santangelo PJ, Rogers KA, Zurla C, Blanchard EL, Gumber S, Strait K, Connor-Stroud F, Schuster DM, Amancha PK, Hong JJ, Byrareddy SN, Hoxie JA, Vidakovic B, Ansari AA, Hunter E, Villinger F. 2015. Whole-body immuno-PET reveals active SIV dynamics in viremic and antiretroviral therapy-treated macaques. Nat Methods 12:427–432. 10.1038/nmeth.3320. 25751144 PMC4425449

[6] Byrareddy SN, Arthos J, Cicala C, Villinger F, Ortiz KT, Little D, Sidell N, Kane MA, Yu J, Jones JW, Santangelo PJ, Zurla C, McKinnon LR, Arnold KB, Woody CE, Walter L, Roos C, Noll A, Van Ryk D, Jelicic K, Cimbro R, Gumber S, Reid MD, Adsay V, Amancha PK, Mayne AE, Parslow TG, Fauci AS, Ansari AA. 2016. Sustained virologic control in SIV^+^ macaques after antiretroviral and alpha4beta7 antibody therapy. Science 354:197–202. 10.1126/science.aag1276. 27738167 PMC5405455

[7] Ventura JD, Beloor J, Allen E, Zhang T, Haugh KA, Uchil PD, Ochsenbauer C, Kieffer C, Kumar P, Hope TJ, Mothes W. 2019. Longitudinal bioluminescent imaging of HIV-1 infection during antiretroviral therapy and treatment interruption in humanized mice. PLoS Pathog 15:e1008161. 10.1371/journal.ppat.1008161. 31805155 PMC6917343

[8] Alberti MO, Jones JJ, Miglietta R, Ding H, Bakshi RK, Edmonds TG, Kappes JC, Ochsenbauer C. 2015. Optimized replicating *Renilla* luciferase reporter HIV-1 utilizing novel internal ribosome entry site elements for native Nef expression and function. AIDS Res Hum Retroviruses 31:1278–1296. 10.1089/aid.2015.0074. 26101895 PMC4663642

[9] Cecilia D, KewalRamani VN, O’Leary J, Volsky B, Nyambi P, Burda S, Xu S, Littman DR, Zolla-Pazner S. 1998. Neutralization profiles of primary human immunodeficiency virus type 1 isolates in the context of coreceptor usage. J Virol 72:6988–6996. 10.1128/JVI.72.9.6988-6996.1998. 9696790 PMC109918

[10] Lee K, Ambrose Z, Martin TD, Oztop I, Mulky A, Julias JG, Vandegraaff N, Baumann JG, Wang R, Yuen W, Takemura T, Shelton K, Taniuchi I, Li Y, Sodroski J, Littman DR, Coffin JM, Hughes SH, Unutmaz D, Engelman A, KewalRamani VN. 2010. Flexible use of nuclear import pathways by HIV-1. Cell Host Microbe 7:221–233. 10.1016/j.chom.2010.02.007. 20227665 PMC2841689

[11] Melody K, Roy CN, Kline C, Cottrell ML, Evans D, Shutt K, Pennings PS, Keele BF, Bility M, Kashuba ADM, Ambrose Z. 2020. Long-acting rilpivirine (RPV) preexposure prophylaxis does not inhibit vaginal transmission of RPV-resistant HIV-1 or select for high-frequency drug resistance in humanized mice. J Virol 94:e01912-19. 10.1128/JVI.01912-19. 31969438 PMC7108851

[12] Takata MA, Goncalves-Carneiro D, Zang TM, Soll SJ, York A, Blanco-Melo D, Bieniasz PD. 2017. CG dinucleotide suppression enables antiviral defence targeting non-self RNA. Nature 550:124–127. 10.1038/nature24039. 28953888 PMC6592701

[13] Weissleder R. 2001. A clearer vision for *in vivo* imaging. Nat Biotechnol 19:316–317. 10.1038/86684. 11283581

[14] Shcherbakova DM, Verkhusha VV. 2013. Near-infrared fluorescent proteins for multicolor *in vivo* imaging. Nat Methods 10:751–754. 10.1038/nmeth.2521. 23770755 PMC3737237

[15] Isomura M, Yamada K, Noguchi K, Nishizono A. 2017. Near-infrared fluorescent protein iRFP720 is optimal for *in vivo* fluorescence imaging of rabies virus infection. J Gen Virol 98:2689–2698. 10.1099/jgv.0.000950. 29039733

[16] Ho YC, Shan L, Hosmane NN, Wang J, Laskey SB, Rosenbloom DI, Lai J, Blankson JN, Siliciano JD, Siliciano RF. 2013. Replication-competent noninduced proviruses in the latent reservoir increase barrier to HIV-1 cure. Cell 155:540–551. 10.1016/j.cell.2013.09.020. 24243014 PMC3896327

[17] Bruner KM, Murray AJ, Pollack RA, Soliman MG, Laskey SB, Capoferri AA, Lai J, Strain MC, Lada SM, Hoh R, Ho YC, Richman DD, Deeks SG, Siliciano JD, Siliciano RF. 2016. Defective proviruses rapidly accumulate during acute HIV-1 infection. Nat Med 22:1043–1049. 10.1038/nm.4156. 27500724 PMC5014606

[18] Imamichi H, Dewar RL, Adelsberger JW, Rehm CA, O’Doherty U, Paxinos EE, Fauci AS, Lane HC. 2016. Defective HIV-1 proviruses produce novel protein-coding RNA species in HIV-infected patients on combination antiretroviral therapy. Proc Natl Acad Sci U S A 113:8783–8788. 10.1073/pnas.1609057113. 27432972 PMC4978246

[19] Pollack RA, Jones RB, Pertea M, Bruner KM, Martin AR, Thomas AS, Capoferri AA, Beg SA, Huang SH, Karandish S, Hao H, Halper-Stromberg E, Yong PC, Kovacs C, Benko E, Siliciano RF, Ho YC. 2017. Defective HIV-1 proviruses are expressed and can be recognized by cytotoxic T lymphocytes, which shape the proviral landscape. Cell Host Microbe 21:494–506. 10.1016/j.chom.2017.03.008. 28407485 PMC5433942

[20] Einkauf KB, Lee GQ, Gao C, Sharaf R, Sun X, Hua S, Chen SM, Jiang C, Lian X, Chowdhury FZ, Rosenberg ES, Chun TW, Li JZ, Yu XG, Lichterfeld M. 2019. Intact HIV-1 proviruses accumulate at distinct chromosomal positions during prolonged antiretroviral therapy. J Clin Invest 129:988–998. 10.1172/JCI124291. 30688658 PMC6391088

[21] Gandhi RT, Cyktor JC, Bosch RJ, Mar H, Laird GM, Martin A, Collier AC, Riddler SA, Macatangay BJ, Rinaldo CR, Eron JJ, Siliciano JD, McMahon DK, Mellors JW, Team AA. 2020. Selective decay of intact HIV-1 proviral DNA on antiretroviral therapy. J Infect Dis 223:225–233. 10.1093/infdis/jiaa532. PMC7857155 32823274

[22] Maldarelli F, Wu X, Su L, Simonetti FR, Shao W, Hill S, Spindler J, Ferris AL, Mellors JW, Kearney MF, Coffin JM, Hughes SH. 2014. HIV latency. Specific HIV integration sites are linked to clonal expansion and persistence of infected cells. Science 345:179–183. 10.1126/science.1254194. 24968937 PMC4262401

[23] Wagner TA, McLaughlin S, Garg K, Cheung CY, Larsen BB, Styrchak S, Huang HC, Edlefsen PT, Mullins JI, Frenkel LM. 2014. HIV latency: proliferation of cells with HIV integrated into cancer genes contributes to persistent infection. Science 345:570–573. 10.1126/science.1256304. 25011556 PMC4230336

[24] Pinzone MR, VanBelzen DJ, Weissman S, Bertuccio MP, Cannon L, Venanzi-Rullo E, Migueles S, Jones RB, Mota T, Joseph SB, Groen K, Pasternak AO, Hwang WT, Sherman B, Vourekas A, Nunnari G, O’Doherty U. 2019. Longitudinal HIV sequencing reveals reservoir expression leading to decay which is obscured by clonal expansion. Nat Commun 10:728. 10.1038/s41467-019-08431-7. 30760706 PMC6374386

[25] Halvas EK, Joseph KW, Brandt LD, Guo S, Sobolewski MD, Jacobs JL, Tumiotto C, Bui JK, Cyktor JC, Keele BF, Morse GD, Bale MJ, Shao W, Kearney MF, Coffin JM, Rausch JW, Wu X, Hughes SH, Mellors JW. 2020. HIV-1 viremia not suppressible by antiretroviral therapy can originate from large T cell clones producing infectious virus. J Clin Invest 130:5847–5857. 10.1172/JCI138099. 33016926 PMC7598056

[26] Mariani R, Rasala BA, Rutter G, Wiegers K, Brandt SM, Krausslich HG, Landau NR. 2001. Mouse-human heterokaryons support efficient human immunodeficiency virus type 1 assembly. J Virol 75:3141–3151. 10.1128/JVI.75.7.3141-3151.2001. 11238841 PMC114108

[27] Hughes SH, Greenhouse JJ, Petropoulos CJ, Sutrave P. 1987. Adaptor plasmids simplify the insertion of foreign DNA into helper-independent retroviral vectors. J Virol 61:3004–3012. 10.1128/JVI.61.10.3004-3012.1987. 3041020 PMC255873

