# Supplementary material for: CG Dinucleotide Removal in Bioluminescent and Fluorescent Reporters Improves HIV-1 Replication and Reporter Gene Expression for Dual Imaging in Humanized Mice
Source: J Virol. 2021 Sep 9;95(19):e00449-21. doi: 10.1128/JVI.00449-21 (PMC8428378; doi:10.1128/JVI.00449-21)
Supplement: Supplemental file 1 — Fig. S1. Download JVI.00449-21-s0001.pdf, PDF file, 0.03 MB [file jvi.00449-21-s0001.pdf]

Figure S1. Sequences of codon-optimized nLuc and iRFP genes.

nLuc

gctagccaccATGGTCTTCACACT**CG**AAGATTT**CG**TTGGGGACTGG**CG**ACAGACAGC**CG**G OG
........................T........T............A..........A.. CO

CTACAACCTGGACCAAGTCCTTGAACAGGGAGGTGTGTCCAGTTTGTTTCAGAATCT**CG**G OG
....A.. CO

GGTGTC**CG**TAACTC**CG**ATCCAAAGGATTGTCCTGAG**CG**GTGAAAATGGGCTGAAGAT**CG**A OG
......T........C....................T....................A.. CO

CATCCATGTCATCATCC**CG**TATGAAGGTCTGAG**CG**G**CG**ACCAAATGGGCCAGAT**CG**AAAA OG
..................A..............T..G.................T..... CO

AATTTTTAAGGTGGTGTACCCTGTGGATGATCATCACTTTAAGGTGATCCTGCACTATGG OG
.... CO

CACACTGGTAAT**CG**A**CG**GGGTTA**CG**C**CG**AACATGAT**CG**ACTATTT**CG**GA**CG**GC**CG**TATGA OG
............T..T........A..C........T........T...A....C..... CO

AGGCAT**CG**C**CG**TGTT**CG**A**CG**GCAAAAAGATCACTGTAACAGGGACCCTGTGGAA**CG**GCAA OG
......A..A.....T..T...................................T..... CO

CAAAATTAT**CG**A**CG**AG**CG**CCTGATCAACCC**CG**A**CG**GCTCCCTGCTGTTC**CG**AGTAACCAT OG
.........A..T...A.A...........A..T...............A.......... CO

CAA**CG**GAGTGAC**CG**GCTGG**CG**GCTGTG**CG**AA**CG**CATTCTGG**CG**TAA OG
...T........A......A.......T...A.G........C... CO

iRFP

gctagccaccATGG**CGCG**TAAGGT**CG**ATCTCACCTCCTG**CG**AT**CGCG**AGC**CG**ATCCACATCC OG
...............TA.A.....A..............T...A.A.....C.......... CO

C**CG**GCAGCATTCAGC**CG**TG**CG**GCTGCCTGCTGGCCTG**CG**A**CGCG**CAGG**CG**GTG**CG**GATCA**CG** OG
.A..............A..T.................T..T..T.....T...A.......C CO

CGCATTA**CG**GAAAATGC**CG**G**CGCG**TTCTTTGGA**CGCG**AAACTC**CGCG**GGT**CG**GTGAGCTACT OG
A.G.....T........T..T..A.........A.A........TA....T........... CO

CGC**CG**ATTACTT**CG**G**CG**AGAC**CG**AAGCCCATG**CG**CTG**CG**CAA**CG**CACTGG**CG**CAGTCCTC**CG** OG
T..T........T..A.....T...........T...A.A..T........T........A. CO

ATCCAAAG**CG**AC**CG**G**CG**CTGATCTT**CG**GTTGG**CGCG**A**CG**GCCTGAC**CG**GC**CG**CACCTT**CG**AC OG
........A....T..A........T......A.A..T........A...A.......T... CO

ATCTCACTGCAT**CG**CCATGA**CG**GTACATCAATCAT**CG**AGTT**CG**AGCCTG**CG**G**CG**GC**CG**AACA OG
............A.......T..............T.....T........A..T..T..... CO

GGC**CG**ACAATC**CG**CTG**CG**GCTGA**CGCG**GCAGATCAT**CGCGCG**CACCAAAGAACTGAAGT**CG**C OG
...T........A...A.......TA..........T..TA.A.................T. CO

T**CG**AAGAGATGGC**CG**CA**CG**GGTGC**CGCG**CTATCTGCAGG**CG**ATGCT**CG**GCTATCAC**CGCG**TG OG
.A...........A...A.......TA.A...........T.....T.........A.A... CO

ATGTTGTAC**CG**CTT**CGCG**GA**CG**A**CG**GCTC**CG**GGATGGTGAT**CG**G**CG**AGG**CG**AAG**CG**CAG**CG**A OG
.........A.G..T..A..T..T.....A...........A..T.....A...A....T.. CO

CCT**CG**AGAGCTTTCT**CG**GTCAGCACTTTC**CG**G**CG**T**CG**CTGGTCC**CG**CAGCAGG**CGCG**GCTAC OG
...A...........A..............A..T..A........A........TA...... CO

TGTACTTGAAGAA**CGCG**ATC**CGCG**TGGTCT**CG**GATT**CGCGCG**GCATCAGCAGC**CG**GAT**CG**TG OG
.............T..T...A.A........A.....AA.A............A....T... CO

CC**CG**AGCA**CG**A**CG**CCTC**CG**G**CG**C**CGCG**CT**CG**ATCTGT**CG**TT**CGCG**CACCTG**CG**CAGCATCTC OG
..A.....T..T.....A..T..A..T..T........C..T..A......A.......... CO

GCCCTGCCATCT**CG**AATTTCTG**CG**GAACATGGG**CG**TCAG**CG**CCT**CG**ATGT**CG**CTGT**CG**ATCA OG
............A.........A..........T.....T.....T.....A.....A.... CO

TCATTGA**CG**GCA**CG**CTATGGGATTGATCATCTGTCATCATTA**CG**AGC**CGCG**TGC**CG**TGC**CG** OG
.......T.....A.............................T.....AA....T.....T CO

ATGG**CG**CAG**CGCG**T**CGCG**GC**CG**AAATGTT**CG**C**CG**ACTTCTTAT**CG**CTGCACTTCAC**CG**C**CG**C OG
.....A...A.A..T..T..A........T..A...........A...........T..T.. CO

CCACCACCAA**CG**CTAA OG
..........A.A... CO
